# Accuracy of Preoperative Scoring Systems for the Prognostication and Treatment of Patients with Spinal Metastases

**DOI:** 10.1155/2017/1320684

**Published:** 2017-08-15

**Authors:** Catherine S. Hibberd, Gerald M. Y. Quan

**Affiliations:** ^1^Department of Surgery, Spinal Biology Research Laboratory, University of Melbourne, Austin Health, Heidelberg, VIC 3084, Australia; ^2^Department of Spinal Surgery and Department of Orthopaedic Surgery, Austin Health, Heidelberg, VIC 3084, Australia

## Abstract

**Background:**

In patients with spinal metastatic disease, survival prognosis is a key consideration in selection for surgery and determining the extent of treatment. Individual survival prediction however remains difficult. We sought to validate the prognostic accuracy of seven preoperative scoring systems.

**Methods:**

61 patients surgically treated for spinal metastases were retrospectively reviewed. Preoperative scores were calculated for Tokuhashi, Revised Tokuhashi, Bauer, Modified Bauer, Sioutos, Tomita, and van der Linden scoring systems. Prognostic value was determined by comparison of predicted and actual survival.

**Results:**

The Revised Tokuhashi and Modified Bauer scoring systems had the best survival predictive accuracy. Rate of agreement for survival prognosis was the greatest for the Modified Bauer score. There was a significant difference in survival of the prognostic groups for all but the van der Linden score, being most significant for the Revised Tokuhashi, Bauer, Modified Bauer, and Tomita scoring systems (*p* ≤ 0.001).

**Conclusion:**

Overall, the scoring systems are accurate at differentiating patients into short-, intermediate-, and long-term survivors. More precise prediction of actual survival is limited and the decision for or against surgery should never be based on survival prognostication alone but should take into account symptoms such as neurological deficit or pain from pathological fracture and instability.

## 1. Introduction

The spine is the commonest site of skeletal metastasis, with widespread occurrence amongst primary breast, lung, prostate, and renal malignancies [1]. Spinal metastases cause significant morbidity to cancer patients, as tumour growth and bony destruction result in pain, pathologic fracture, and cord compression, which impair the ambulatory ability and worsen quality of life. Despite the advances in nonoperative treatment of spinal metastases, surgery remains the only method for immediate mechanical stabilisation and spinal cord decompression and, in appropriately selected patients, improves pain, function, and quality of life for the duration of the patients' remaining lifetime [2–7]. Survival prognosis is one of the most important considerations in selecting patients for surgery, in order to determine which patients will benefit from surgery and to guide the magnitude of surgery to be performed. In predicted long-term survivors, more extensive spinal cord and nerve decompression, gross tumour resection, and instrumented fixation should be performed in order to minimise the risk of local tumour recurrence and need for further procedures. Conversely, in patients with poor survival prognosis, surgery may be contraindicated, or limited surgery may be offered. Factors related to survival include histologic tumour type, extent of disease, pathological fracture, neurologic deficit, and functional status [1, 8, 9]. Accuracy in individual prognostication however remains difficult. Specific to patients with spinal metastases, a number of scoring systems aimed at prognostication and treatment decision-making, including guidelines for extent of surgery, are presented in the literature [8, 10–14]. However, these scoring systems differ in both the clinical and the radiological parameters assessed, weighted scoring, and proposed treatment strategies and accuracy reported in validation studies [11, 15–18] (see Table 1).  Table 2 summarises the study particulars for each scoring system (cohort number, treatment regime, and cohort type).

Tokuhashi et al. performed a retrospective analysis of 64 patients surgically treated for spinal metastases to devise a preoperative prognostic scoring system, comprised of six parameters, to guide surgical intervention [19]. This scoring system was later revised to take into account the strong influence of primary tumour type on survival by scoring this parameter on a maximum of 5 points [12]. The Revised Tokuhashi is the only scoring system to clearly define both predicted prognosis and treatment strategy for those seeking to evaluate their own patients.

Bauer and Wedin developed a prognostication model for survival after surgery for spinal and extremity metastases, identifying five positive prognostic variables of approximate equal weighting: absence of visceral metastases, absence of pathologic fracture, not primary lung cancer, solitary skeletal metastases, and primary tumour of breast, kidney, lymphoma, or myeloma [10]. In this study of 241 cancer patients, pathological fracture was in fact associated with lower survival in the extremity group only [10], prompting Leithner et al. to propose a Modified Bauer score that excludes pathologic fracture as a prognostic variable [11]. The proposed treatment according to the prognostic group follows stepwise progression from nonoperative management to extensive excisional procedures [11].

Sioutos et al. determined three negative prognostic factors with a compounding adverse effect on overall survival: preoperative leg strength 0/5–3/5, lung or colon cancer, and multiple vertebral body disease [8]. Radical surgery is not recommended for patients with two or more negative prognostic factors (predicted survival less than 6 months) given minimal potential for benefit; however, no further recommendations regarding treatment choice, such as for those without any negative prognostic factors, are proposed for this scoring system [8].

Tomita et al. conducted a two-stage study to develop a prognostic scoring system for surgical treatment of patients with spinal metastases [13]. Phase 1 involved a retrospective review of the treatment of 67 patients with spinal metastases to evaluate the predictive value of the scoring system [13]. 61 patients were prospectively enrolled in Phase 2 whereby treatment was primarily determined according to prognostic score; the majority reported to have achieved the desired local tumour control, pain relief, and neurologic improvement [13].

The van der Linden scoring system differs from others discussed with regard to disease severity of the study cohort and subsequent approach to treatment [14]. A review of 342 patients with painful spinal metastases involved in a randomised trial of radiotherapy identified three significant predictors of survival: site of primary tumour, visceral metastases, and functional status [14]. Of the three proposed prognostic groups, surgery is only considered appropriate for patients with the best prognosis in the setting of persistent pain despite radiotherapy, or when spinal cord tolerance after radiotherapy has been reached [14].

Patients with metastatic spinal cancer have a median life expectancy of 1 to 2 years, and the goal of treatment whether nonoperative or operative should be for symptom palliation, that is, to maintain or restore spinal stability, to reduce pain, and to improve or prevent neurologic deterioration in order to maintain function and quality of life [1, 3, 5, 20]. Individual survival prediction however remains difficult, and the reported accuracy of prognostic scoring systems presented in the literature is variable, prompting us to perform a validation of the survival accuracy of seven preoperative prognostic scoring systems for patients with spinal metastases.

## 2. Methods

72 consecutive patients who had undergone surgical intervention for metastatic spinal disease at a single institution between January 2010 and July 2013 were identified. Inclusion criteria were greater than 12-month postoperative follow-up (unless deceased earlier) and completeness of medical record data and imaging. 11 patients were excluded due to inadequate follow-up; 61 patients were subsequently eligible for inclusion in the analysis. Indications for surgery were intractable pain or neurological deficit due to pathological fracture, spinal instability with or without metastatic epidural spinal cord or nerve root compression, and minimum estimated survival greater than 3 months as determined by the treating oncology team. The survival period was calculated from the date of operation until the date of death or last follow-up. Clinical features of the cohort are detailed in Table 3.

Preoperative clinical and radiological parameters were extracted from the digital medical record and imaging database, and procedural details were obtained from operation notes. In a retrospective manner, preoperative scores for each patient were calculated by a single clinician using the following scoring systems: Tokuhashi [19], Revised Tokuhashi [12], Bauer [10], Modified Bauer [11], Sioutos [8], Tomita [13], and van der Linden [14].

The prognostic value of each scoring system was evaluated by comparison of predicted and actual survival. Where prognostic group survival was not specified by the scoring system, prognosis was determined from reported survival rates of the cohort in the original publication.

### 2.1. Statistical Analysis

The data were stored in an Excel database (Microsoft Corp., Redmond, WA, USA) and analysed using IBM SPSS Statistics Standard Version 21.0 and Stata Version 12 IC software. Survival predictive accuracy was calculated by comparison of predicted prognosis according to the scoring system with actual survival period after treatment, reported as percentage correct. Crosstabs analysis and calculation of weighted Cohen's kappa were used to measure the rate of agreement between predicted and actual survival for each scoring system. The kappa statistic measures agreement between two or more observers or observations, taking into account the fact that agreement or disagreement will sometimes occur simply by chance; a kappa of 1 indicates perfect agreement, whereas a kappa of 0 indicates agreement equivalent to chance.

Kaplan-Meier survival curves were generated for each scoring system to determine survival characteristics and compare prognostic group survival outcomes of each scoring system. Log-rank test was used to determine significance in survival of the prognostic groups within each scoring system, the results of which were verified with the Generalised Wilcoxon test. *p* value of <0.05 was considered statistically significant.

## 3. Results

61 patients (9 females, 52 males) with at least 12-month postoperative follow-up, unless deceased earlier, were included in the survival analysis. Average age at operation was 62.4 years (range: 25–85), and all patients underwent spinal decompression and/or instrumented stabilisation surgery (further detailed in Table 3). 42 patients were deceased, for whom median survival was 102 days (range: 10 days–1.6 years). For those alive at the last follow-up, median follow-up time was 1.9 years (range: 1–3.4 years).

### 3.1. Survival Predictive Accuracy

The Revised Tokuhashi and Modified Bauer scoring systems had the greatest survival predictive accuracy between predicted prognosis and actual postoperative survival (72% and 69% resp.), and both performed better than their original versions: 59% and 72% survival predictive accuracy for the Original and Revised Tokuhashi scoring systems, respectively, and 62% and 69% survival predictive accuracy for the Bauer and Modified Bauer scoring systems, respectively (see Table 4). The Tomita scoring system was the worst performed with only 51% correct survival prediction.

### 3.2. Rate of Agreement

The Modified Bauer score had the highest rate of agreement for survival prognosis with a weighted Cohen's kappa score of 0.50 (95% CI: [0.28–0.70]) indicating moderate agreement (Table 4). The Revised Tokuhashi, Bauer, and Tomita scoring systems were also found to have a moderate rate of agreement between predicted and actual survival, weighted Cohen's kappa scores of 0.41, 0.41, and 0.43, respectively. In keeping with results of survival predictive accuracy, both the Revised Tokuhashi and the Modified Bauer scores had a greater rate of agreement than their original versions, weighted Cohen's kappa scores of 0.39 and 0.41 for the Original and Revised Tokuhashi scoring systems and 0.41 and 0.50 for the Bauer and Modified Bauer scoring systems, respectively. The van der Linden scoring system was the worst performed, with only a “slight” rate of agreement (weighted Cohen's kappa = 0.17) between predicted and actual survival.

### 3.3. Survival Distribution (Log-Rank Test)

**Figure 1:**
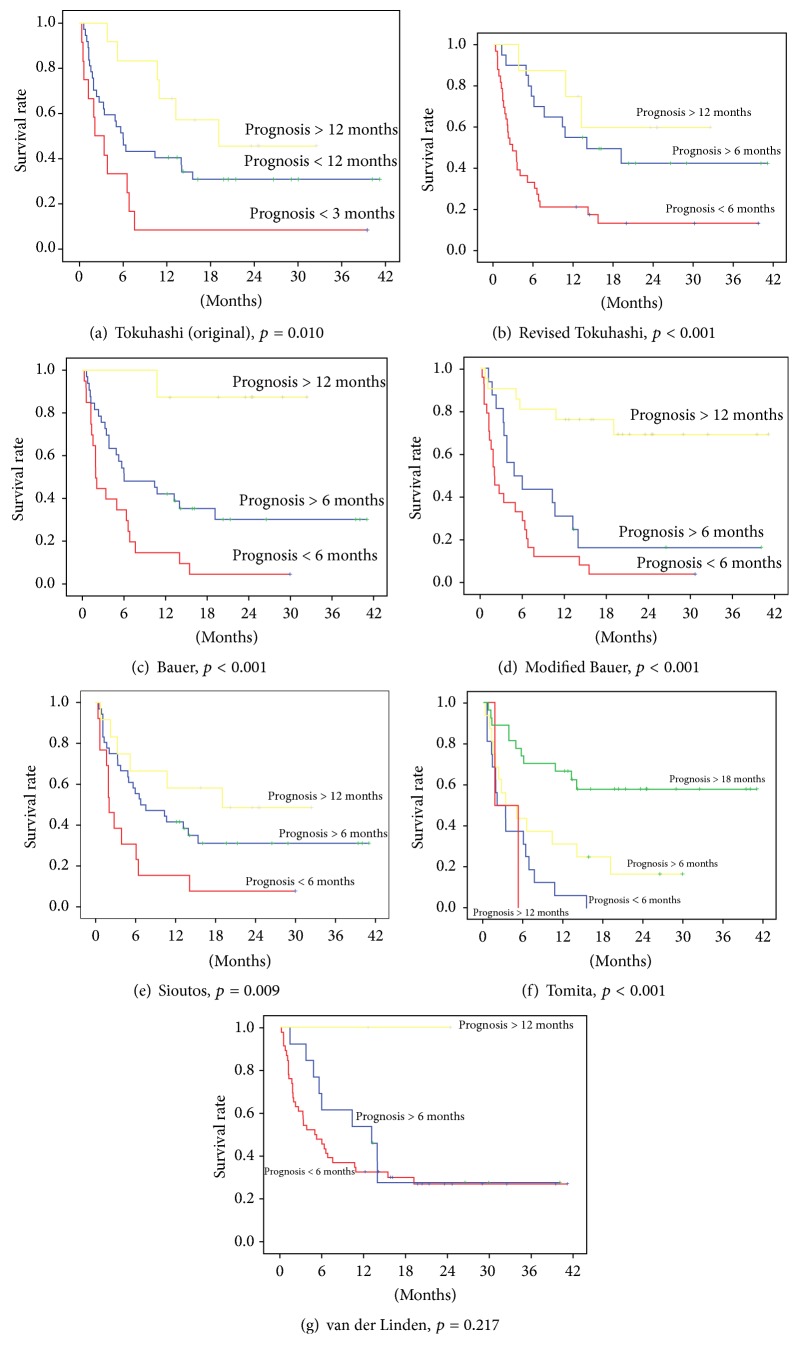
Kaplan-Meier survival curves (log-rank test) for the prognostic groups of the seven scoring systems.

Kaplan-Meier survival curves were generated for each scoring system to compare actual survival outcomes of the prognostic groups (Figure 1). The results of the log-rank test to assess statistical significance in life expectancy of the prognostic groups within each scoring system are presented in Figure 1. Log-rank test demonstrated a statistically significant difference in survival of the prognostic groups for all but the van der Linden scoring system. Survival distribution between prognostic groups was most significant for the Revised Tokuhashi, Bauer, Modified Bauer, and Tomita scoring systems (*p* ≤ 0.001).

## 4. Discussion

Specific to patients with spinal metastases, a number of scoring systems aimed at prognostication and treatment decision-making, including guidelines for extent of surgery, are presented in the literature [9, 11, 12]. The majority of these scoring systems were developed by retrospective assessment of data from relatively small cohorts (<100 patients) incorporating patients of a diverse range of primary cancer types, stages of disease, and treatment or intervention received. Furthermore, although the original authors of the scoring systems demonstrated accuracy of survival prognostication, independent analysis has not always replicated similar findings [11, 15, 17]. In addition, there are few validation or comparison studies of the prognostic scoring systems presented in the literature [11, 15–18].

Individually, the scoring systems are accurate at differentiating patients into short-, intermediate-, and long-term survivors. From the Kaplan-Meier curves generated for each scoring system, log-rank test demonstrated a statistically significant difference in survival of the prognostic groups for all but the van der Linden scoring system. Indeed, survival distribution between prognostic groups was most significant for the Revised Tokuhashi, Bauer, Modified Bauer, and Tomita scoring systems (*p* ≤ 0.001). Our findings are in accordance with that of Leithner et al., who retrospectively analysed 69 patients treated surgically for spinal metastases and determined the Revised Tokuhashi, Tomita, Bauer, Modified Bauer, and van der Linden scoring systems to provide a statistically significant difference in survival of the prognostic groups [11]. The Modified Bauer score, which excludes pathologic fracture as a negative prognostic variable, was determined to be of the best association with survival (log-rank test, *p* < 0.001) [11]. In a further survival analysis of preoperative scoring systems, Wibmer et al. found the Modified Bauer score to provide the most reliable results and the Bauer and Modified Bauer to be the only scoring systems for which a significant difference in the life expectancy of the three prognostic groups (good, moderate, and poor) was identified [9]. More precise prediction of actual survival however is limited. Survival predictive accuracy comparing actual to predicted survival period after treatment was found to be between 50 and 70% for the seven scoring systems. The revised Tokuhashi score had the highest survival predictive accuracy (72%), and therefore while approximately two-thirds of cases are predicted correctly, the remaining one-third of cases are predicted incorrectly. Furthermore, the rate of agreement between predicted and actual survival was found to be “moderate” for only four of the scoring systems, with the remainder considered “slight” or “fair.”

Tokuhashi et al. performed a combined retrospective and prospective analysis of their revised scoring system and demonstrated greater than 80% consistency between predicted prognosis and actual survival [12]. However, Pointillart et al. found neither the Original nor the Revised Tokuhashi scoring systems to be reliable in predicting survival, with overall less than 60% accuracy [5]. Quraishi et al. also found modest overall predictive value of 66% of the Tokuhashi score in over 200 patients with metastatic spinal cord compression [21]. Furthermore, Ulmar et al. report low reliability in survival prediction of the Original and Revised Tokuhashi scoring systems, particularly for those with less than 1-year predicted survival [16]. In a study of risk factors affecting survival in patients above 60 years of age with spinal metastases, Liang et al. identified the accuracy of the Original Tokuhashi score to vary considerably; scores for poor and good prognostic groups accurately predicted survival in 78% and 82% of patients, respectively; however, scores for the moderate prognostic group were accurate in only 41% of patients [22]. Although a statistically significant difference in the survival between prognostic groups (log-rank test) of the Original and Revised Tokuhashi scoring systems was identified, there was only a moderate rate of agreement between predicted and actual survival. Therefore, we are in agreement with others [16, 22] that while the Original and Revised Tokuhashi scoring systems are useful for prognostic grouping, their individual survival prognostication value is limited.

The Sioutos scoring system scored poorly for both survival predictive accuracy and rate of agreement. Our findings are in accordance with that of Leithner et al. who found no correlation between predicted and real survival for the Sioutos score [11]. The Sioutos scoring system is perhaps too simplified with only two outcome measures (absent or present) for the three negative prognostic variables scored, rather than the weighted scoring common to other scoring systems. The Tomita scoring system was the worst performed for survival predictive accuracy. This is in accordance with Ulmar et al., who did not identify significance between prognosticated and real survival in their analysis of the Tomita scoring system [17].

Overall, the scoring systems adequately differentiate patient survival groups, and thus the individual parameters assessed indeed influence survival. Table 1 provides a summary of the scoring systems with respect to the parameters assessed: primary tumour type, number of extraspinal metastases, and presence of visceral metastases are the most common parameters included in the scoring systems. Primary tumour type is considered one of the most important prognostic factors for survival in patients with spinal metastases [5, 23]. Tumour biology determines growth rate, radiosensitivity, and response to chemotherapy, which influence life expectancy and treatment outcomes [8]. Spinal metastases of lung cancer in particular are associated with poor survival, compared to that of breast cancer and multiple myeloma [5, 8, 10, 12–14]. In revising their original scoring system, Tokuhashi et al. identified that patients with a primary lesion of lung, bladder, or upper gastrointestinal origin had an average survival of less than 6 months and were therefore assigned 0 points [12]. In contrast, patients with a primary lesion of thyroid, breast, prostate, or carcinoid tumour survived on average for greater than 1 year and were subsequently assigned 5 points [12].

Tomita et al. include primary tumour growth rate rather than primary tumour type per se as a prognostic variable, whereby slow growth rate tumours are considered the most favourable for survival [13]. It has been suggested that primary tumour type may indirectly affect survival through influence on other prognostic factors such as the presence of extraspinal and visceral metastases, which are associated with the activity of the primary disease [23]. Extraspinal bone metastases have been identified as a significant independent prognostic factor of survival following multivariate analysis of patients with both spinal [23] and skeletal [24] metastases and are included as a prognostic variable in all but the Sioutos and van der Linden scoring systems. However, in an analysis of prognostic scoring systems for spinal metastases, Leithner et al. did not identify the number of extraspinal bone metastases (none versus one or more) as a significant prognostic factor for survival [11]. Metastasis to major internal organs (lungs, liver, kidney, and brain) is considered a significant prognostic factor for survival in patients with spinal metastases [10, 11, 13, 14, 19, 23], as well as in those with skeletal metastases [24]. It is suggested that the presence of visceral metastases reflects the aggressiveness of tumour growth and metastatic tumour load, which would typically result in decreased general condition of the patient [10, 11]. Functional decline, often secondary to effects of skeletal and visceral metastases, is considered an additional measure of tumour disease burden [11, 13]. Conversely to this, in a prospective analysis of 142 preoperative candidates for spinal metastases, Pointillart et al. did not identify the presence of visceral metastases to affect survival [5]. Furthermore, Sioutos et al. found that although patients with visceral metastases had a shorter postoperative survival (median survival of 12.0 versus 7.5 months), this did not reach statistical significance and was therefore not included in their prognostic scoring system [8].

The scoring systems included in our analysis use survival prognostication as a means to determine treatment, including whether conservative or surgical management is indicated and the extent of surgical intervention. Overall, we identified that these scoring systems had, at best, a moderate rate of agreement between predicted and actual survival and up to 70% survival predictive accuracy. If these classification systems and their prognostication are being used as surgical guidelines in treatment decision-making, then approximately one-third of patients may receive inadequate or excessive surgery. Previous studies of the application of prognostic scoring systems focus on survival prognostication as the primary outcome in determining validity, yet it is reiterated that estimated survival is not the only determinant of treatment strategy, and other key factors such as mechanical instability and symptomatic cord compression are also imperative [3, 11, 25]. The Spinal Instability Neoplastic Score (SINS) is an example of a classification system, based upon patients' symptoms and radiographic criteria, that aims to predict spine stability of neoplastic lesions in order to identify patients who may benefit from surgical consultation [26].

Despite the advances in noninvasive treatments, surgery remains an indispensable treatment option for patients with spinal metastases. Although there is a considerable amount of literature on survival prognostication as a means for determining treatment in patients with spinal metastases, comparable knowledge and understanding of the significance and prediction of factors necessitating surgery, such as fracture and cord compression, is lacking. In agreement with Leithner et al. [11], we are of the opinion that the decision for or against surgery should never be based on survival prognostication alone but should take into account symptoms such as neurological deficit or pain from pathological fracture and instability.

## 5. Conclusion

Survival prognosis is a key consideration in selecting patients for surgery; however, accuracy in individual prognostication and prediction of long-term survivors who will benefit most from surgery and short-term survivors who would not benefit from surgery remain a difficult challenge. Specific to patients with spinal metastases, there are a number of scoring systems aimed at prognostication and treatment decision-making, including guidelines for extent of surgery. We found the Revised Tokuhashi, Bauer, Modified Bauer, and Tomita scoring systems to be the best performed for survival analysis across three domains: survival predictive accuracy, rate of agreement, and survival distribution. These scoring systems however are more useful for the stratification of survival prognostic groups, rather than their survival predictive accuracy per se, and should be used as an estimate only in guiding treatment planning.

## Figures and Tables

**Table 1:** Preoperative prognostic scoring systems for patients with spinal metastases.

Scoring system	Prognostic variables (score)	Total score	Predicted prognosis (months)	Treatment strategy
Tokuhashi	(i) General condition (KPS) (0, 1, 2) (ii) Extraspinal bone mets (0, 1, 2) (iii) Number of vertebral body mets (0, 1, 2) (iv) Major organ metastasis (0, 1, 2) (v) Primary site of cancer (0, 1, 2) (vi) Spinal cord palsy (Frankel) (0, 1, 2) *Total score: 0*–*12*	≤5 6–8 9–12	<3 ≤12 ≥12	Palliative surgery (not specified) Excisional surgery

Revised Tokuhashi	(i) General condition (KPS) (0, 1, 2) (ii) Extraspinal metastasis (0, 1, 2) (iii) Number of vertebral body mets (0, 1, 2) (iv) Major organ metastasis (0, 1, 2) (v) Primary site of cancer (0–5) (vi) Palsy (Frankel) (0, 1, 2) *Total score: 0*–*15*	0–8 9–11 12–15	<6 ≥6 ≥12	Conservative Palliative surgery: SC decompression and stabilisation Excisional surgery: en bloc resection, curettage, and stabilisation

Bauer	Positive prognostic factors (i) Pathologic fracture (0, 1) (ii) No visceral metastases (0, 1) (iii) Solitary skeletal metastases (0, 1) (iv) No lung cancer (0, 1) (v) Primary cancer: breast/kidney/myeloma/lymphoma (0, 1) *Total score: 0*–*5*	0-1 2-3 4-5	<6 >6 >12	Conservative Palliative surgery Excisional surgery

Modified Bauer	(i) No visceral metastases (0, 1) (ii) Solitary skeletal metastases (0, 1) (iii) No lung cancer (0, 1) (iv) Primary tumour: breast/kidney/myeloma/lymphoma (0, 1) *Total score: 0*–*4*	0-1 2 3-4	<6 >6 >12	Conservative Dorsal surgery (palliative) Ventral-dorsal surgery (excisional)

Sioutos	Negative prognostic factors	0	>12	Excisional surgery
	(i) Preoperative leg strength 0/5–3/5 (0, 1)	1	>6	Palliative surgery
	(ii) Lung or colon cancer (0, 1)	2	<6	Conservative: surgery not recommended
	(iii) Multiple vertebral body disease (0, 1) *Total score: 0*–*3*	3	<6	

Tomita	(i) Primary tumour growth rate (1, 2, 4) (ii) Visceral metastases (0, 2, 4) (iii) Bone metastases (1, 2) *Total score: 2–12*	2-3 4-5 6-7 8–10	>18 >12 >6 <6	Wide or marginal excision Marginal or intralesional excision Palliative surgery (decompression/stabilisation) Conservative: supportive care

van der Linden	(i) General condition (KPS) (0, 1, 2) (ii) Primary tumour type (0, 1, 2, 3) (iii) Visceral metastases (0, 1) *Total score: 0–6*	0–3 (Group A) 4-5 (Group B) 6 (Group C)	<6 >6 >12	Radiotherapy Radiotherapy Surgery, only if imminent

**Table 2:** Study details for preoperative prognostic scoring systems.

Scoring system (year)	Cohort number	Treatment	Study type
Tokuhashi (1990)	64	Surgery	Retrospective
Revised Tokuhashi (2005)	246	Surgery (164), conservative (82)	118 patients, prospective; total 246 retrospective
Bauer (1995)	153 extremity metastases, 88 spinal metastases	Surgery	Prospective
Modified Bauer (2008)	69	Surgery	Retrospective
Sioutos (1995)	109	Surgery	Retrospective
Tomita (2001)	Phase 1: 67 Phase 2: 61	Phase 1: not specified Phase 2: surgery (52), supportive care (9)	Phase 1: retrospective Phase 2: prospective
van der Linden (2005)	342	Radiotherapy (Harrington class 1 & 2 painful spinal metastasis)	Retrospective

**Table 3:** Patients' demographics and clinical features.

		Number of cases	%
Site of tumour	Cervical spine	8	13
	Thoracic spine	41	67
	Lumbar	12	20

Primary tumour	Lung	10	16.5
	Myeloma	10	16.5
	Prostate	10	16.5
	Breast	5	8
	Melanoma	5	8
	Colorectal	4	7
	Bladder	4	7
	Renal	3	5
	Unknown primary	3	5
	Lymphoma	2	3
	HCC	2	3
	Oesophageal	1	1.5
	Cholangiocarcinoma	1	1.5
	Neuroendocrine	1	1.5

Tumour effect	Pathologic fracture	48	79
	Instability	51	84
	Cord compression	43	71
	Neural compression	42	69
	Cord or neural compression	53	87
	Neurologic deficit	27	44

Surgical procedure	Anterior corpectomy/stabilisation	Cervical (6), thoracic (2)	
	Posterior decompression alone	Thoracic (2)	
	Posterior stabilisation alone	Cervical (1), thoracic (3), lumbar (3)	
	Posterior decompression/stabilisation	Thoracic (34), lumbar (8)	
	Ant. & post. decompression/stabilisation	Cervical (1), thoracic (1)	

**Table 4:** Results of survival predictive accuracy and rate of agreement between predicted and actual survival for each scoring system.

Scoring system	Survival predictive accuracy		Rate of agreement		
	Correct survival prediction^a^	% correct	Weighted Cohen's kappa^b^	95% CI	Interpretation of agreement
Original Tokuhashi	36	59%	0.39	0.21–0.56	Fair
Revised Tokuhashi	44	72%	0.41	0.24–0.62	Moderate
Bauer	38	62%	0.41	0.23–0.58	Moderate
Modified Bauer	42	69%	0.50	0.28–0.70	Moderate
Sioutos	37	61%	0.26	0.03–0.45	Fair
Tomita	30	51%	0.43	0.23–0.63	Moderate
van der Linden	36	59%	0.17	0.02–0.36	Slight

^a^ *N* of 61 patients. ^b^Kappa of 1 indicates perfect agreement; a kappa of 0 indicates agreement equivalent to chance; <0: less than chance agreement; 0.01–0.20: slight agreement; 0.21–0.40: fair agreement; 0.41–0.60: moderate agreement; 0.61–0.80: substantial agreement; 0.81–0.99: almost perfect agreement.
